# Occurrence of Chordoid Glioma With Sodium Ion Metabolism Disorder 5 Years After Meningioma Surgery and Whole-Exome Sequencing: A Case Report and Literature Review

**DOI:** 10.3389/fgene.2021.617575

**Published:** 2021-05-10

**Authors:** Mei Zhang, Baofeng Xu, Chang Li, Ziwei Liu, Yuanyuan Gao, Yuming Song, Rui Liu

**Affiliations:** ^1^Department of VIP Unit, China-Japan Union Hospital of Jilin University, Changchun, China; ^2^Department of Neurosurgery, First Hospital of Jilin University, Changchun, China; ^3^Department of Endocrinology, China-Japan Union Hospital of Jilin University, Changchun, China

**Keywords:** fibrous meningioma, chordoid glioma, third ventricle, hyponatremia, hypernatremia, whole-exome sequencing, *PRKCA*^D463H^

## Abstract

Chordoid glioma (CG), a rare slow-growing brain tumor, mainly occurs in the region of the third ventricle. Although its degree of malignancy is relatively low, its clinical prognosis is poor due to obscure clinical manifestations and the particular growing position. Currently, gross total resection is the best available method for treatment of CG. However, the tumor is located in the deep structure of the brain and close to neurovascular structure so it is difficult to remove completely. This study reported a case of CG of the third ventricle 5 years after surgery of right frontal parietal fibrous meningioma, accompanied with peri and post-operative sodium ion metabolism disorder. Whole-exome sequencing (WES) revealed 25 gene mutations shared by meningioma and CG. In addition, the *PRKCA*^*D*463*H*^ CG marker gene mutation also existed in this patient. We reviewed the latest literature on this rare brain tumor, summarized its clinical manifestations, imaging and pathological characteristics, and discussed the mechanism related to its occurrence and the reasons for sodium ion disorder.

## Introduction

Fibrous meningioma and chordoid glioma (CG), two primary intracranial tumors of different origins, have disparate pathological manifestations. As a subtype of meningiomas, fibrous meningioma is often found in the elderly and middle-aged individuals (Louis et al., 2007). Surgical resection is an effective treatment linked to less disease recurrence and metastasis compared with vs. other modalities. CG was originally thought to be a variant type of chordoid meningioma, as the immunophenotyping of the tumor became known, it was formally named by Brat et al. (1998). At present, the histogenesis and anatomical origin of CG is not completely clear. The World Health Organization (WHO) has classified it as an indeterminate Grade II neuroepithelial tumor (Louis et al., 2016), which often occurs in the third ventricle, as well as the temporoparietal occipital lobe, cerebellum and hypothalamus (Jain et al., 2008; Jin et al., 2010; Yang et al., 2019). The tumor usually has no particular clinical manifestations, and is mainly related to compression of adjacent tissues, such as hypothalamus, pituitary, thalamus, optic chiasma, etc. Histopathologically, CG presents chordoma-like clusters, composed of glial fibrillary acidic protein (GFAP)-positive tumor cells embedded in the mucus matrix, and exhibits the characteristics of lymphocyte and plasma cell infiltration (Ki et al., 2016). Despite the low histological score, the clinical prognosis of CG is poor, its particular growth location complicates complete resection, and adjuvant treatment provides uncertain results and aggressive behaviors have been reported in some of the literature (Ampie et al., 2015; Ki et al., 2016). We reported a case of third ventricle CG, which occurred 5 years after the total resection of fibrous meningioma, and the peri and post-operative ion disorder may be related to endocrine metabolic diseases. Literature search did not yield previous reports of co-occurrence of these two solid tumors in a single patient. We reviewed the clinical manifestations, imaging, and pathological features of the tumor, and focused on the pathogenesis of the tumor and causes of abnormal sodium ion metabolism to deepen the clinical understanding of CG. We present the following case in accordance with the CARE reporting checklist (Gagnier et al., 2013).

## Case Description

### Initial Presentation and Operation

**Figure 1:**
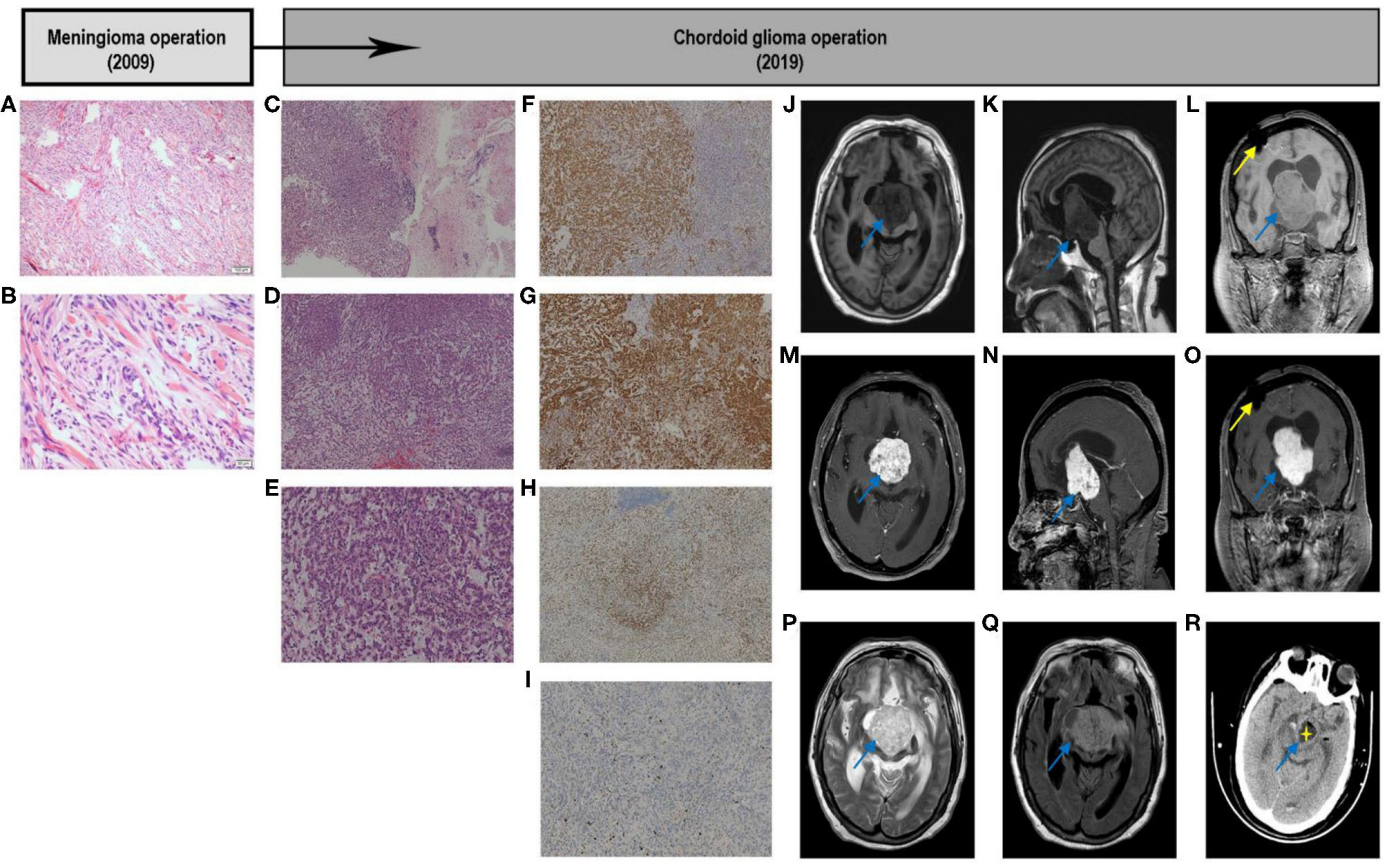
Graphic summary of case. Fibrous meningioma. Hematoxylin–eosin. Low-magnification images showing that tumor cells are ovoid and arranged in bundles and swirls **(A)**. High-magnification images showing that tumor cells are arranged in bundles and swirls, with focal fibrous proliferation. The tumor cells are ovoid and short fusiform, with a non-mitotic phase **(B)**. Chordoid Glioma. Hematoxylin–eosin. Low-magnification images showing tumor tissue on the left and brain tissue on the right, and part of the boundary between the two is unclear. Tumor cells are arranged in chords, clusters, and crack, with mucus, lymphocytes, and plasma cells in the stroma **(C)**. The tumor cells are arranged in cords of epithelioid and nest, with a mucus, lymphocytes and plasma cells in the stroma **(D)**. The tumor cells are fairly uniform, with plenty of cytoplasm, eosinophilic, and medium nucleus, most were in a non-mitotic phase, with mucus, lymphocytes, and plasma cells in the stroma **(E)**. Immunohistochemical. Immunostaining GFAP demonstrated cytoplasmatic diffuse and strong expression in tumor cells **(F)**; CD34 showed cytoplasmatic positivity in tumor cells **(G)**; TTF**-**1 showed nuclear positivity in most cells **(H)**; Ki-67 showed slight nuclear positivity in cells and a low proliferation index 5% **(I)**. Original magnification: **(A,C,D,F–H)** ×40; **(E,I)** ×100; **(B)** ×200. GFAP, glial fibrillary acidic protein; TTF**-**1, transcription termination factor 1. Image examination. T1-weighted axial **(J)**, sagittal **(K)**, and coronal **(L)** scans showing that the discontinuity of the right parietal bone, local patchy artifacts, the bilateral lateral ventricle, and the third ventricle were obviously dilated; the scans also show, clumpy long T1 signal shadows in the right lateral ventricle and third ventricle, with clear boundaries. The size was ~ 4.0 × 4.5 × 5.5 cm, the small piece of the long T1 signal shadow can be observed locally, and the boundary was unclear. The boundary between the lower part of the lesion and brainstem was unclear, and the brainstem was obviously compressed. Enhanced scan showed that the tumor exhibited heterogeneous enhancement **(M–O)**. The tumor showed a high-low mixed signal on the T2-weighted axial scan **(P)**. T2-Flair axial scan showed a slightly hyperintense signal shadow on the tumor **(Q)**. Early post-operative CT scan showed that most of the tumor tissue was removed. The removed part was filled with cerebrospinal fluid, and part of the tumor wall remained **(R)**. CT, computed tomography; Flair, Fluid-attenuated inversion recovery.

A 53-year-old female was hospitalized in June 2009 due to sudden syncope episodes. Magnetic resonance imaging (MRI) revealed the presence of a right frontal parietal lobe anterior central gyrus tumor (diameter: 2.5 × 3.0 × 3.0 cm). The tumor was predominantly isointense on T1-weighted (T1WI) and T2-weighted (T2WI), and enhancement was homogeneous. The clinical diagnosis was meningioma. The tumor was completely resected microsurgically through the right frontal top approach, and a post-operative pathological diagnosis of meningioma was reached (fibrous, WHO I) (Figures 1A,B). The patient recovered well following surgery, with no complications, and did not receive radiotherapy or chemotherapy. During a routine follow-up in 2014, an interpeduncular cistern- occupying lesion was detected, however, treatment was not administered.

### Second Presentation, Operation, and Adjuvant Therapy

In November 2019, the patient was hospitalized with fever caused by aspiration pneumonia. Over the past 5 years, the patient had been bedridden, her state of consciousness had gradually deteriorated and she was experiencing drowsiness. MRI showed an irregular tumor in front of the third ventricle, invading the right ventricle and compressing the brainstem. T1W1 showed a slightly lower signal, and T2W1 showed a slightly increasing signal, with heterogeneous contrast enhancement, accompanied by obstructive hydrocephalus (Figures 1J–Q). Preoperative laboratory test results indicated severe hyponatremia (<125 mmol/L). After adjusting the sodium ions to the normal range, the tumor was microsurgically removed via the right ventricle approach to the third ventricle. During the operation, the tumor capsule was intact, gray-red, soft, and the blood flow was moderate, fully removed in the capsule. The tumor tightly adhered to the midbrain and pontine, and part of the tumor envelope remained on the surface of the basilar artery. Thus, subtotal resection was performed (Figure 1R), and post-operative pathological examination confirmed the CG of the third ventricle (WHO II) (Figures 1C–E). Histologically, part of the tumor tissue and brain tissue boundaries were unclear. Immunohistochemical analysis was characterized by strong reaction to GFAP, and there was expression of CD34, transcription termination factor-1(TTF-1), vimentin, and epithelial membrane antigen (Figures 1F–J). The Ki-67 proliferation index was lower than normal (5.0%) (Figure 1I).

Following the operation, a series of complications (i.e., ion disorder, infection, and respiratory failure) occurred. The metabolic disorder of sodium ion was particularly obvious, with hypernatremia (155.1 mmol/L) in the early stage which became hyponatremia (123.9 mmol/L) at 1 week after surgery (Supplementary Figure 1). After continuous renal replacement therapy, solution replenishment, and down-regulation of hydrocortisone, the levels of sodium ion stabilized and the patient's state of consciousness improved slightly 50 days after surgery (Supplementary Table 1).

### Genetic Analysis

To investigate the pathogenesis of the two different primary intracranial tumors, we applied whole-exome sequencing on the two tumors to analyze all variants annotated with in-house pipeline. The patient's available tumor sample (formalin-fixed paraffin-embedded [FFPE] tumor samples) were processed for testing. DNA was extracted from FFPE blocks of tumor tissues obtained from the patient using the QIAamp DNA FFPE Tissue Kit (QIAGEN, 56404, Germany). Sequencing libraries were prepared from genomic DNA, and targeted enrichment of target gene fragments with probes. Captured libraries were sequenced reads on an Illumina HiSeq 4000 instrument (HiSeq, America), and duplicate sequencing reads were computationally removed. The data were analyzed according to the absence of a control group. Population polymorphisms (database of 1,000 groups) and mutations not present in the exon region were removed. The sites were predicted as harmful mutations by software, mutations with abundance of 40**–**60% and 90**–**100% were further eliminated.

**Figure 2:**
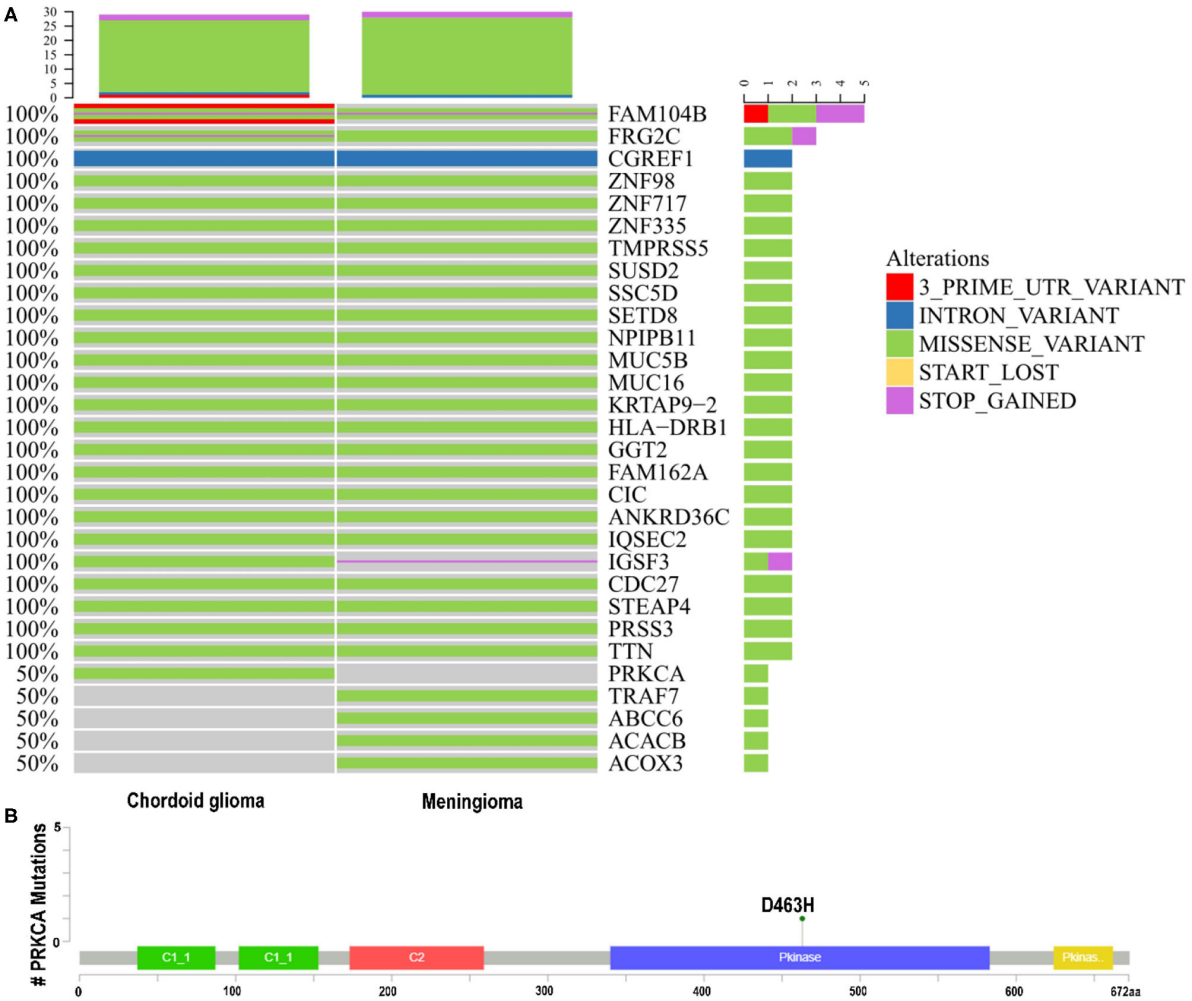
**(A,B)** Whole-exome sequencing. Known driver gene mutation analysis indicated the missense mutations of *TRAF7* in the meningioma and *PRKCA* ^*D*463*H*^ in the chordoid glioma. *PRKCA*, protein kinase C alpha; *TRAF7*, TNF receptor associated factor 7.

Among 321 meningioma gene mutations and 94 CG gene mutations screened by analysis, 25 shared gene mutations with potential significance were found by bio-information analysis (*CGREF1, NPIPB11, CDC27, STEAP4, MUC5B, FAM162A, TTN, HLA-DRB1, ZNF98, IGSF3, SUSD2, GGT2, ZNF717, FAM104B, SSC5D, TMPRSS5, CIC, ANKRD36C, MUC16, PRSS3, ZNF335, KRTAP9-2, FRG2C, IQSEC2, SETD8*). Consistent with previously reported cases, the meningioma in this patient harbored a mutation on chromosome 16. A C>T transversion mutation in the *TRAF7* gene was identified, causing a c.1991C>T, p.T664I substitution (reference transcript NM_032271.2). CG harbored a mutation on chromosome 17, a G>C transversion mutation in the *protein kinase C alpha (PRKCA)* gene was identified, causing a c.1387G>C, p.D463H substitution (reference transcript NM_002737.2) (Figure 2).

Categorize the detected variants based on their clinical impact according to the guidelines for interpretation and reporting of sequence variants in cancer (Li et al., 2017). Base on the results of multiple small studies (Goode et al., 2018; Shai et al., 2018), *PRKCA*^*D*463*H*^ is a specific mutant gene of CG, which is of great significance for diagnosis. In Tier-Based reporting, it is classified as Tier II variables: variables with potential clinical significance (variables with level C diagnostic / predictive significance). There has been no report about the coexistence of two solid tumors, CG and meningioma, so there is no report on the 25 mutation genes shared by them. In Tier-Based reporting, it is classified as Tier III Variants: Variants of Unknown Significance.

## Discussion

CG, a rare solid brain tumor with both glial and chordoid features, often occurs in adult females (male-to-female ratio, 1:2). Due to the morbidity of the disease and the limitation of test samples, the genetic basis and pathogenesis of CG only partly understood. Based on the research of ultrastructure and immunohistochemistry, several hypotheses (e.g., glial origin, ependymal origin, and organum vasculosum at the lamina terminalis) were proposed (Cenacchi et al., 2001; Sato et al., 2003; Leeds et al., 2006; Bielle et al., 2015). Studies found a recurrent *D463H* missense mutation in *PRKCA* in CGs, which localizes in the kinase domain of the encoded protein kinase C alpha (PKCα) (Goode et al., 2018; Shai et al., 2018). In addition, other genes test were no alterations identified (Table 1), suggesting that CGs are also genetically distinct from the majority of ependymomas, diffuse lower-grade gliomas, and astrocytomas (Capper et al., 2018; Goode et al., 2018; Shai et al., 2018). Since the *PRKCA*^*D*463*H*^ mutation is highly specific for CG, it appears to genetically redefine CG of the third ventricle and distinguish it from the types of brain tumors that have been investigated. Our patient also had *PRKCA*^*D*463*H*^ mutation. While the precise mechanism by which this *PRKCA* mutation drives glioma remains to be elucidated, it increases the mitogen-activated protein kinase (MAPK) signaling pathway, eukaryotic initiation factor 2 (EIF2) pathway, phosphoinositide 3-kinase-AKT-mechanistic target of rapamycin kinase (PI3K-AKT-mTOR), and Ras signaling pathway activation driving the development of CG (Supplementary Figure 2). Thus, this mutation provides new insights into the genetic mechanism of CG, and provides potential new targets for treatment. Different from previously reported cases of CGs, in our patient, CG merged with another solid brain tumor and combined with abnormal peri and post-operative sodium ion metabolism disorder. To the best of our knowledge, this is the first case of this kind. CG is rarely accompanied by other components. Thus far, only four cases of CG coexisting with other different histological components have been reported (Supplementary Table 2). The co-occurrence of two tumors may be attributed to changes in the microenvironment caused by the presence of the first tumor (Suh et al., 2003; Poyuran et al., 2016; Yao et al., 2017). Whole-exome sequencing analysis showed that there were 25 shared mutations; this indicated that the rare successively evolutionary relationships between two intracranial tumors may have a certain correlation. However, compared with the common mutation genes of meningioma and CG, there were no screened same gene mutations. At the same time, due to the lack of a negative control, the occurrence of two kinds of tumors may have an independent clonal origin, this is worthy of further investigation.

**Table 1:** Case reports involving chordoid glioma related-gene detection.

**References**	**Number of test cases**	**Genes**
Reifenberger et al. (1999)	4	*TP53, CDKN2A, EGFR, CDK4, MDM2* (–)
Bielle et al. (2015)	16	*IDH1 R132, IDH2 R172, BRAF^*V*600*E*^* (–)
Shai et al. (2018)	13	*PRKCA ^*D*463*H*^* (+) *NF2, RELA, IDH1, IDH2, TP53, ATRX, TERT, CIC, FUBP1, NOTCH1, TSC1, TSC2* (–)
Goode et al. (2018)	17	*PRKCA ^*D*463*H*^* (+)
Capper et al. (2018)	12	*MDM4, MYCN, GLI2, FGFR3/TACC3, PDGFRA, TERT, MYB, CDK6, KIAA1549/BRAF, FGRR1/TACC1, MYBL1, MYC, PTCH1, PTEN, MGMT, CCND1, CCND2, CKD4, MDM2, RB1, TP53, NF1, PPM1D, C19MC, AMARCB1, NF2* (–)

The clinical manifestations are mainly related to the location, size, and growth mode of the tumor, without specificity. Part of the symptoms (e.g., headaches, dizziness, insomnia, fatigue, blurred vision, mental and memory disorders, etc.) are due to the compression of adjacent tissues. Preoperative radiographic diagnosis of tumor may be difficult; some CGs mimic other types of lesions (particularly chordoid meningiomas) which are prone to misdiagnosis. Moreover, it also necessary to distinguish CGs from tumors that were inherent or extrinsically extending to the third ventricle. According to computed tomography scans, the tumor was hyperdense to gray matter with homogeneous enhancement. MRI showed T1W1 imaging hypo-to isointensity, T2W1 imaging iso-to hyperintensity, and enhancement was homogeneous or heterogeneous (Bongetta et al., 2015). In some patients, MRI suggests that vasogenic edema and hydrocephalus are relatively special characteristic features, which have great significance for promoting prospective diagnosis (Shinohara et al., 2018). The present case also exhibited these characteristics. However, the definitive diagnosis of neoplasms mainly depends on histology and immunohistochemistry results. Histologically, CG is composed of chords of epithelioid cells within mucinous stroma containing lymphocytes and plasma cells. Tumor immunopositivity for GFAP, epithelial membrane antigen, CD34, cytokeratin, S100, and vimentin are commonly present. The Ki-67 proliferative indices were <5%. As a transcription factor involved in ventral forebrain development, TTF-1 is expressed in most third ventricular CG. Although not specific, it is considered as a marker for differentiation from other saddle and third ventricular tumors (Bielle et al., 2015).

Repeated laboratory testing of the patient prior to surgery indicated hyponatremia (sodium 124.6 mmol/L; plasma osmolality 269.96 mOsm/kg). After the exclusion of pseudo hyponatremia, adrenocortical hypofunction, and hypothyroidism, we considered that the preoperative hyponatremia may have been partially related to the syndrome of inappropriate antidiuretic hormone (SIADH), which can be secondary to CG. In 2016, Calanchini et al. reported a case presenting with hyponatremia partly due to SIADH, which was secondary to third ventricle CG (Calanchini et al., 2016). SIADH has occasionally been reported prior to brain tumor surgery, presenting with hyponatremia and seizures in response to fluid restriction. Tumor masses may result in inappropriate release of ADH through direct mechanical stimulation and/or osmotic receptor/ischemic changes in ADH secretory neurons (Edate and Albanese, 2015; Cuesta et al., 2016). Our patient was unable to assess the clinical changes caused by hyponatremia due to changes in the state of consciousness. Symptoms caused by neuroendocrine dysfunction may be relatively rare in CGs; a few cases with amenorrhea, hypothyroidism, diabetes insipidus and SIADH have been reported (Brat et al., 1998; Raizer et al., 2003; Kurian et al., 2005; Dziurzynski et al., 2009; Ni et al., 2013; Edate and Albanese, 2015; Calanchini et al., 2016; Cuesta et al., 2016; Danilowicz et al., 2018) (Supplementary Table 3). Central diabetes insipidus, SIADH and cerebral salt wasting syndrome are the main reasons for craniocerebral surgery in the treatment of sodium metabolism imbalance. Nevertheless, intravenous fluids, administration of dehydration drugs, and total parenteral nutrition may also cause (Brimioulle et al., 2008; Overgaard-Steensen and Ring, 2013; Ball and Iqbal, 2015; Edate and Albanese, 2015; Cuesta et al., 2016; Cui et al., 2019) (Table 2). Coexistence of these conditions complicates diagnosis and management. Similar to most neurological tumors, CG is also associated with post-operative sodium disturbance. Good management is important for improving the prognosis of patients. We considered the possibility of temporary diabetes insipidus after the operation. In addition, the patient also had inability to eat and drink, infection, and fever, and received dehydration drugs, which complicated the diagnosis. The management of post-operative fluid and electrolyte disorders following CG surgery is challenging. Maintenance of sodium homeostasis is essential for better treatment response and patient survival. Only close multidisciplinary collaboration can achieve optimal treatment of this rare and complex disease.

**Table 2:** Causes of sodium homeostasis disorders after craniocerebral surgery.

	**Etiology**	**Clinical manifestations**	**Main diagnostic indicators**	**Treatment**
**Hyponatremia (central)**				
CSWS	Inappropriate NP secretion, Sympathetic inhibition	Polyuria, Hypovolemia	Central nervous system diseases; Sodium: <135mmol/L; Plasma osmolality : <275mOsm/kg; Urine sodium: >20mmol/L or 80mmol/d; Urine osmolality: >300 mOsm/kg;	Water and salt supplementation, Mineralocorticoid
SIADH	Endogenous ADH secretion increased	Decreased urine output, Thirst, Hypervolemia	Sodium: <135mmol/L; Plasma osmolality: <275mOsm/kg; Urine sodium: >20mmol/L; Urine osmolality: >100 mOsm/kg;	Fluid restriction, Sodium supplemental, Vaptans
Adenohypophysis dysfunction	Surgical or radiation injury, TSH, ACTH↓	Low metabolic syndrome, Fatigue, Weakness Loss of appetite, Hypotension, Hypoglycemia Etc.	TSH, FT3, FT4↓; ACTH, F↓;	Hormone replacement therapy
**Hypernatremia (central)**				
CDI	ADH partially or completely lacking	Thirsty, Polydipsia, Polyuria	Plasma osmolality: >300 mOsm/kg; Urine output: > 40–50 mL/kg/day; Urine osmolality: <300 mOsm/kg; Urine/plasma osmolality ratio <1;	d-DDAVP
Hypothalamic ADI	ADH deficiency, Lack of thirst mechanism	Polyuria, Dehydration	Lack of thirst sensation	Fixed fluids ingestion, d-DDAVP administration
**Hyponatremia (non-central)**				
Related to fluids Drugs	Decreased intake, Increased excretion	Vomiting, Diarrhea, Insufficient sodium supplement with dehydrating drugs, etc.	Clinical manifestations, Fluid intake, Fluid output, Medication, etc.	Oral or intravenous sodium supplementation, Remove the original cause
**Hypernatremia (non-central)**				
Related to fluids Drugs	Recessive water loss, Hypotonic body fluid loss	Fever, Water restriction, Dehydrating drugs, Vomiting, Diarrhea, etc.	Clinical manifestations, Fluid intake, Fluid output, Medication, etc.	Limit sodium intake, Supplement glucose solution, Hemodialysis

*ACTH, adrenocorticotropic hormone; ADI, adipic central diabetes insipidus; CDI, central diabetes insipidus; CSWS, cerebral salt wasting syndrome; NP, natriuretic peptides; SIADH, syndrome of inappropriate antidiuretic hormone; TSH, thyroid-stimulating hormone*.

Complete tumor resection is currently the gold standard in the treatment of CG (Kobayashi et al., 2013). The traditional surgical method for CG resection is transcranial, mainly including transcallosal, transcortical, and trans-lamina terminalis approaches; the choice of approach depends on the location and growth of the tumor. Retrospective findings of trans-lamina terminalis may be associated with lower rates of post-operative mortality and complications compared with other approaches; however, there is limited data to support this hypothesis (Liu et al., 2011; Ki et al., 2016; Carrasco-Moro et al., 2018). In 2016, a CG patient with vision and visual field changes underwent surgery through the expanded objective transnasal approach for complete removal of the tumor (Zeinalizadeh et al., 2016). Compared with the transcrania approach, the endoscopic transnasal approach can provide excellent visualization of the undersurface of the optic chiasm and optic nerves. The success of the expanded endoscopic transnasal approach for the treatment of CG in the third ventricle has open up new horizons and provides a new alternative approach. Clinically, owing the location of tumor growth and post-operative complications, only select patients can undergo gross total resection. Conventional radiotherapy, stereotactic radiosurgery, and Gamma Knife radiosurgery can be performed in patients in whom complete resection of the tumor is not possible. The optimal treatment strategy for CG is controversial; whether tumor biopsy/partial resection and subsequent adjuvant therapy is a better treatment method warrants further investigation (Kobayashi et al., 2013).

The occurrence of two primary intracranial tumors without a history of radiation therapy or genetic disease is a very rare event. The shared gene mutations found by WES provided evidences to link the origin of CG to other brain tumors. Due to the long-term preservation of tumor specimens and the loss of negative control during follow-up testing, the quality of data may be affected to some extent. The interpretation and annotation of the variants were limited. In this article, we put forward a new hypothesis for the origin of these two different tumors, but further studies are needed to understand the biological behavior of this rare event. Owing to the limitation of the patient's consciousness and some objective factors, we were unable to conduct more in-depth inspection and endocrine experiments on the sodium ion disorder, but ADH may play an important role in sodium metabolism disorder. When combining surgery for ion disorder related to third ventricle tumors and post-operative management, treating physicians should pay attention to the comprehensive evaluation and management of the neurosecretory system.

## Data Availability Statement

The original contributions presented in the study are included in the article/Supplementary Material, further inquiries can be directed to the corresponding author/s.

## Ethics Statement

The studies involving human participants were reviewed and approved by Ethics committee of China-Japan Union Hospital of Jilin University. The patients/participants provided their written informed consent to participate in this study. Written informed consent was obtained from the individual(s) for the publication of any potentially identifiable images or data included in this article.

## Author Contributions

MZ collected patient clinical data and was a major contributor in writing the manuscript. BX, YS, and RL were in charge of analyzing and interpreting the patient data, and revising the draft critically for important intellectual content. CL, ZL, and YG were responsible for collecting the clinical data. All authors read and approved the final manuscript.

## Conflict of Interest

The authors declare that the research was conducted in the absence of any commercial or financial relationships that could be construed as a potential conflict of interest.

## Supplementary Material

Supplementary Figure 1 Sodium concentration. Preoperative hyponatremia. Post-operative hyponatremia and hypernatremia alternated **(A)**. Urine specific gravity. Post-operative urine volume was 3–4 L/day (basically balanced inflow and outflow) with low specific gravity **(B)**.

Supplementary Figure 2 Mechanism of chordoid glioma. *EIF2*, eukaryotic initiation factor 2; *ERK*, extracellular signal-regulated kinase; *mTOR*, mechanistic target of rapamycin kinase; *PI3K*, phosphoinositide 3-kinase; *PRKCA*, protein kinase C alpha.

Supplementary Table 1 Laboratory test results at baseline, and post-operative 30 and 50 days.

Supplementary Table 2 Reported cases of collision of chordoid glioma.

Supplementary Table 3 Endocrine dysfunction symptoms.
