# Supplementary figures and images for: Occurrence of Chordoid Glioma With Sodium Ion Metabolism Disorder 5 Years After Meningioma Surgery and Whole-Exome Sequencing: A Case Report and Literature Review
Source: Front Genet. 2021 May 10;12:617575. doi: 10.3389/fgene.2021.617575 (PMC8143433; doi:10.3389/fgene.2021.617575)

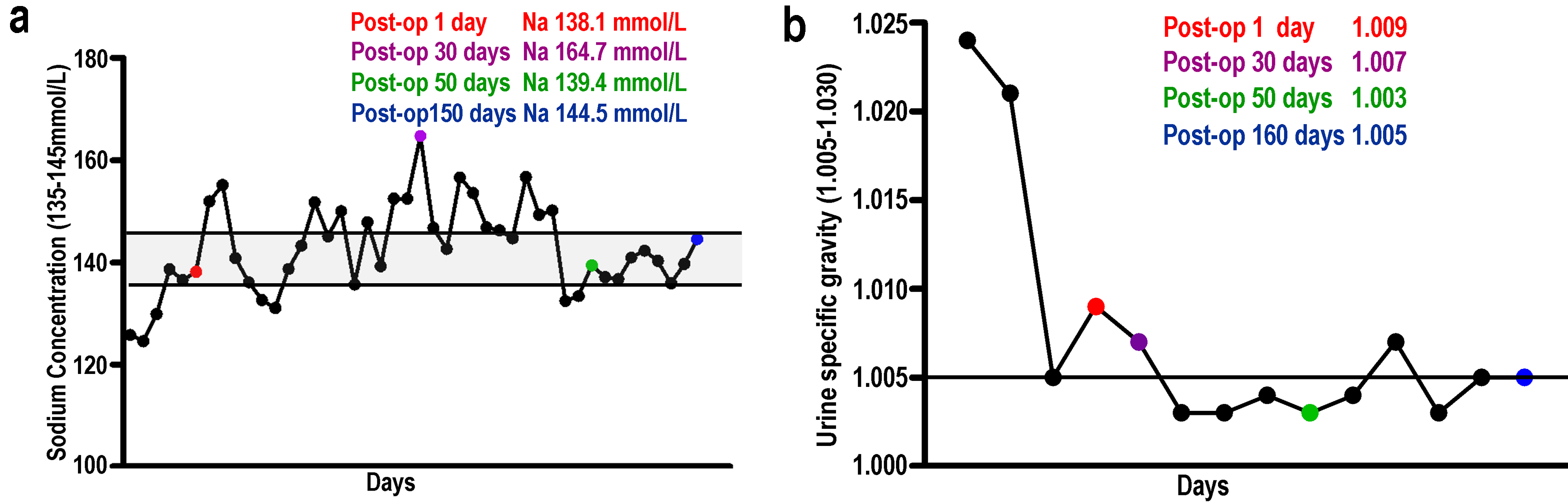

Supplement: Supplementary Figure 1 — Sodium concentration. Preoperative hyponatremia. Post-operative hyponatremia and hypernatremia alternated (A). Urine specific gravity. Post-operative urine volume was 3–4 L/day (basically balanced inflow and outflow) with low specific gravity (B). [file Image_1.TIF]

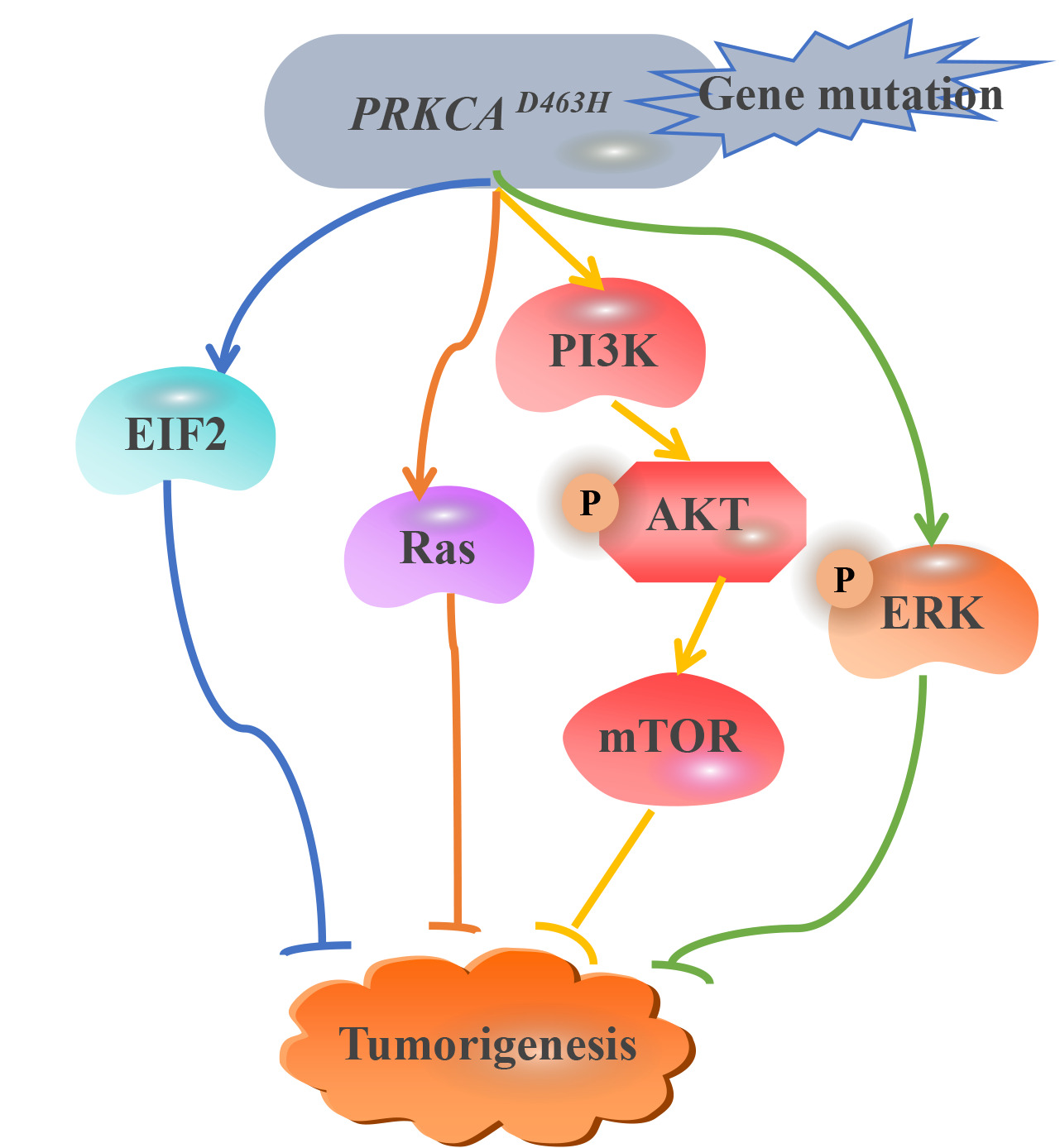

Supplement: Supplementary Figure 2 — Mechanism of chordoid glioma. EIF2, eukaryotic initiation factor 2; ERK, extracellular signal-regulated kinase; mTOR, mechanistic target of rapamycin kinase; PI3K, phosphoinositide 3-kinase; PRKCA, protein kinase C alpha. [file Image_2.tif]
