# Supplementary material for: Occurrence of Chordoid Glioma With Sodium Ion Metabolism Disorder 5 Years After Meningioma Surgery and Whole-Exome Sequencing: A Case Report and Literature Review
Source: Front Genet. 2021 May 10;12:617575. doi: 10.3389/fgene.2021.617575 (PMC8143433; doi:10.3389/fgene.2021.617575)
Supplement: Supplementary Table 1 — Laboratory test results at baseline, and post-operative 30 and 50 days. [file Table_1.docx]

**Supplementary Table 1 Laboratory test results at baseline, and postoperative 30 and 50 days.**

|  | Baseline | Postoperative 30 days | Postoperative 50 days |
| --- | --- | --- | --- |
| Sodium (135–145 mmol/L) | 124.6 | 164.7 | 139.4 |
| Potassium (3.5–5.0 mmol/L) | 4.25 | 2.78 | 4.14 |
| GLU (3.9–6.1 mmol/L) | 7.94 | 4.42 | 5.65 |
| Plasma osmolality (278–295 mOsm/kg) | 269.96 | 343.98 | 295.56 |
| Urine sodium (40–220 mmol/24h) |  | 42.9 | 49.28 |
| Urine specific gravity (1.005–1.300) | 1.024 | 1.007 | 1.003 |
| Creatinine (48–100 umol/L) | 62.58 | 102.56 | 99.35 |
| eGFR (ml/min/1.73 m2) | 90.96 | 50.06 | 52.02 |
| Urea (2.8–7.6 mmol/L) | 4.32 | 4.6 | 2.83 |
| TSH (0.372–4.94 mIU/L) | 0.811 | 0.983 | 1.46 |
| FT3 (3.1–6.8 pmol/L) | 3.05 | 2.44 | 2.64 |
| FT4 (12–22 pmol/L) | 15.3 | 10.8 | 13.7 |
| ACTH 08:00 h (7.2–63.4 pg/mL) | 17.2 | <1.00 | 1.39 |
| Serum cortisol 08:00 h (139–690 nmol/L) | 540.1 | 46.7 |  |
| Serum cortisol 16:00 h (85.3–459.6nmol/L) | 333 | 88.8 |  |
| Serum cortisol 24:00 h (36.5–186.9 nmol/L) | 175 | 40.9 |  |
| Urinary free cortisol (53.2–789.4 nmol/24h) |  | 531.08 | 255.09 |
| PRL (108.8–557.1 mIU/L) | 622.1 | 1439 | 1170.9 |
| GH (0.02–5.42 ng/mL) | <0.02 | <0.02 | <0.02 |
| FSH (menopause 26.72–133.41 IU/L) | 4.2 | 1.7 | 1.8 |
| LH (menopause 5.16–61.99 IU/L) | 1.5 | 0.2 | 0.3 |
| Estradiol (menopause 1–102.8 pmol/L) |  | <37 | 80 |

ACTH, adrenocorticotropic hormone; FSH, follicle-stimulating hormone; GH, growth hormone; LH, luteinizing hormone; PRL, prolactin; TSH, thyroid-stimulating hormone.
