# Supplementary material for: Occurrence of Chordoid Glioma With Sodium Ion Metabolism Disorder 5 Years After Meningioma Surgery and Whole-Exome Sequencing: A Case Report and Literature Review
Source: Front Genet. 2021 May 10;12:617575. doi: 10.3389/fgene.2021.617575 (PMC8143433; doi:10.3389/fgene.2021.617575)
Supplement: Supplementary Table 2 — Reported cases of collision of chordoid glioma. [file Table_2.DOCX]

**Supplementary Table 2 Reported cases of collision of chordoid glioma.**

| Author, year | Age | Sex | Tumor location | Another histological component |
| --- | --- | --- | --- | --- |
| Y. L. Suh et al., 2003 | 48 | Female | Sellar Suprasellar | Rathke’s cleft cyst |
| R. Poyuran et al., 2016 | 45 | Female | Hypothalamus Third ventricle | Epidermoid cyst |
| K. Yao et al., 2017 | 45 | Female | Anterior third ventricle Suprasellar | Rosai–Dorfman |
|  | 38 | Male | Third ventricle | Rosai–Dorfman |
