# Supplementary material for: Occurrence of Chordoid Glioma With Sodium Ion Metabolism Disorder 5 Years After Meningioma Surgery and Whole-Exome Sequencing: A Case Report and Literature Review
Source: Front Genet. 2021 May 10;12:617575. doi: 10.3389/fgene.2021.617575 (PMC8143433; doi:10.3389/fgene.2021.617575)
Supplement: Supplementary Table 3 — Endocrine dysfunction symptoms. [file Table_3.DOCX]

**Supplementary Table 3 Endocrine dysfunction symptoms.**

| Author, year | Age | Sex | Symptoms |
| --- | --- | --- | --- |
| D. J. Brat et al., 1998 | 56 | Female | Hypothyroidism DI |
|  | 31 | Female | Hypothyroidism |
|  | 35 | Female | Amenorrhea |
| J. J. Raizer et al., 2003 | 57 | Female | DI |
| K. M. Kurian et al., 2005 | 32 | Female | Amenorrhea |
| K. Dziurzynski et al., 2009 | 41 | Female | DI |
| H. C. Ni et al., 2012 | 35 | Female | Amenorrhea |
| K. Danilowicz et al., 2016 | 18 | Female | Amenorrhea |
|  | 46 | Female | Amenorrhea Galactorrhea |
| M. Calanchini et al., 2016 | 48 | Female | SIADH |

DI, diabetes insipidus; SIADH, syndrome of inappropriate antidiuretic hormone.
